# Supplementary material for: Reversible proliferative arrest induced by rapid depletion of RNase MRP
Source: Nat Commun. 2025 Jun 18;16:5342. doi: 10.1038/s41467-025-60471-4 (PMC12177063; doi:10.1038/s41467-025-60471-4)
Supplement: Supplementary file 1 — Supplementary Information [file 41467_2025_60471_MOESM1_ESM.pdf]

Reversible proliferative arrest induced by rapid depletion of RNase MRP

Yuan Liu, Shiyang He, Kawon Pyo, Reuben Franklin, Ibrahim B. Maaz, Chen Cai, Kriti Shah, Sihem Cheloufi, William F. Marzluff, Jernej Murn

Supplementary Information

6 Supplementary Figures

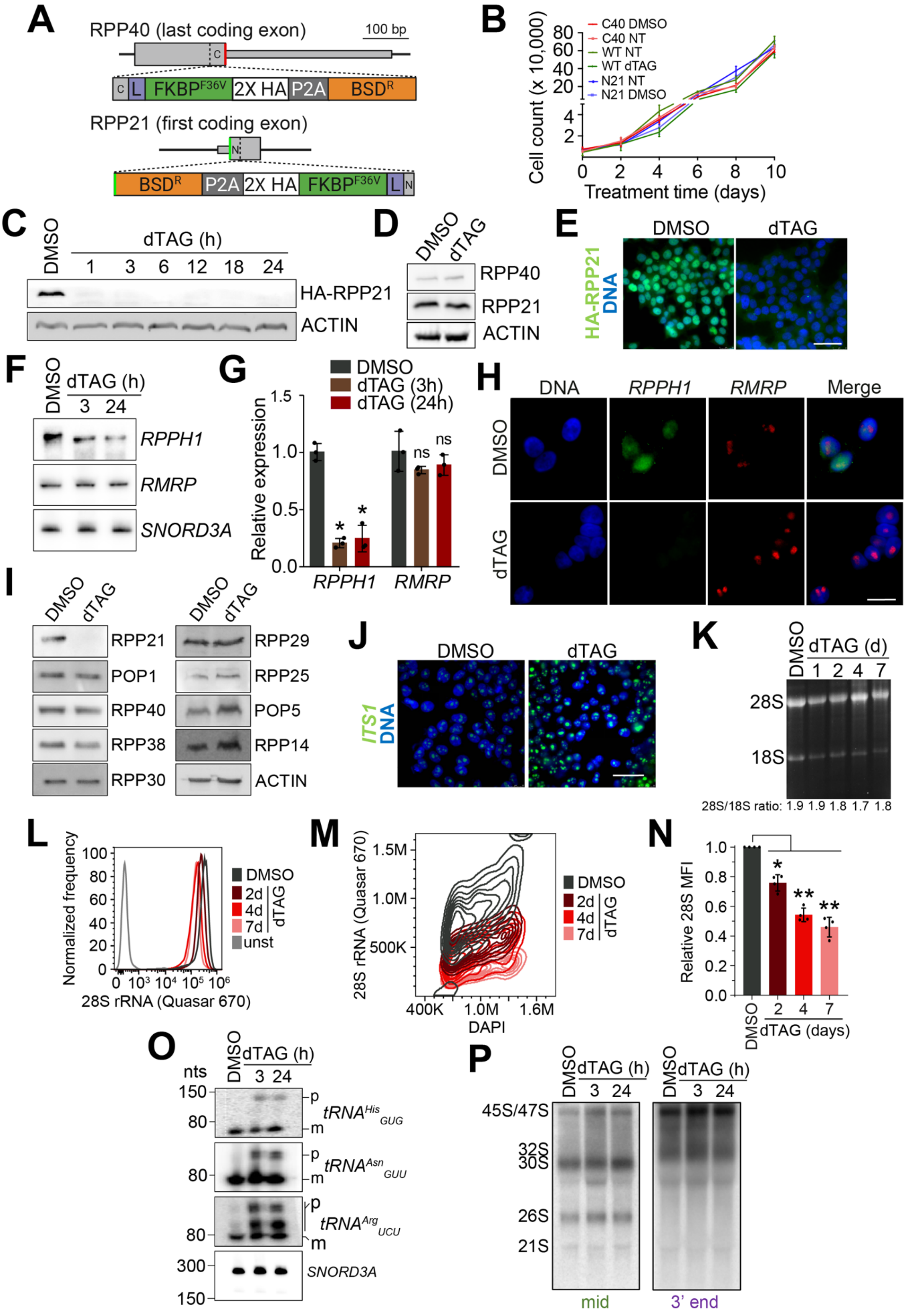
A
RPP40 (last coding exon)
100 bp
C L FKBP^F36V 2X HA P2A BSD^R
RPP21 (first coding exon)
BSD^R P2A 2X HA FKBP^F36V L N
B
C40 DMSO
C40 NT
WT NT
WT dTAG
N21 NT
N21 DMSO
Cell count (x 10,000)
Treatment time (days)
C
DMSO
dTAG (h)
1 3 6 12 18 24
HA-RPP21
ACTIN
D
DMSO dTAG
RPP40
RPP21
ACTIN
E
DMSO dTAG
HA-RPP21 DNA
F
DMSO dTAG (h) 3 24
RPPH1
RMRP
SNORD3A
G
DMSO
dTAG (3h)
dTAG (24h)
Relative expression
RPPH1 RMRP
ns
H
DNA RPPH1 RMRP Merge
DMSO
dTAG
I
DMSO dTAG
RPP21 POP1 RPP40 RPP38 RPP30
RPP29 RPP25 POP5 RPP14 ACTIN
J
DMSO dTAG
ITS1 DNA
K
DMSO dTAG (d) 1 2 4 7
28S
18S
28S/18S ratio: 1.9 1.9 1.8 1.7 1.8
L
Normalized frequency
28S rRNA (Quasar 670)
DMSO 2d 4d 7d dTAG unst
M
28S rRNA (Quasar 670)
DAPI
DMSO 2d 4d 7d dTAG
N
Relative 28S MFI
DMSO dTAG (days)
O
nts
DMSO dTAG (h) 3 24
tRNA^His GUG
tRNA^Asn GUU
tRNA^Arg UCU
SNORD3A
P
DMSO dTAG (h) 3 24
45S/47S
32S
30S
26S
21S
mid
3' end

Supplementary Fig. 1 | Inducible depletion of RNase P alone or RNases P and MRP in human cells. A Schematic illustrating the strategy, originally described in Nabet *et al.* [1], for the composition of the degron tag (FKBP^{F36V})-containing knock-in cassettes, and the location of their CRISPR/Cas9-mediated insertion homozygously into the *RPP40* (top) and *RPP21* (bottom) loci of HEK293T cells to create the C40 and N21 cell lines, respectively. The same strategy was also used to generate the *RPP40* degron-tagged HCT116 cell line. BSD^R, blasticidin resistance gene; L, linker; C and N, addback codons. **B** Growth curves of non-treated (NT) or dTAG-treated parental HEK293T cells (WT) and their derived C40 and N21 cells, DMSO-treated or non-treated. Data are shown as mean ± SD (n = 3 biological replicates). **C** Immunoblot analysis of the endogenous (tagged) RPP21 using anti-HA antibody in lysates of N21 cells treated with DMSO or dTAG for the indicated time periods. ACTIN serves as a loading control (n = 3). **D** Immunoblot analysis of DMSO- or dTAG-treated HEK293T cells using anti-RPP40 and anti-RPP21 antibodies. ACTIN serves as a loading control (n = 3). **E** Representative image of N21 cells treated with DMSO or dTAG for 3 h and stained for DNA content (blue) and HA-RPP21 (using anti-HA antibody; green). Scale bar, 50 µm. **F** Northern analysis of *RPPH1* and *RMRP* from N21 cells treated with DMSO or dTAG, as indicated. *SNORD3A* serves as a loading control (n = 3). **G** Relative quantification by qPCR of RNA samples as in **F** (n = 3 biological replicates). Data are shown as mean ± SD. *, $p < 0.005$ (two-tailed Student's t test); exact p values in Source Data file. **H** RNA FISH of *RPPH1* and *RMRP* in N21 cells treated with DMSO or dTAG for 3 h. Scale bar, 20 µm. **I** Immunoblot analysis of RNase P and MRP protein subunits in lysates of N21 cells treated with DMSO or dTAG for 24 h (n = 3). **J** Representative image of N21 cells treated with DMSO or dTAG for 24h h and stained for DNA content (blue) and the ITS1 segment of rRNA (green). Scale bar, 50 µm. **K** Relative levels of 28S and 18S rRNA in total RNA isolated from C40 cells treated with either DMSO or dTAG for the indicated periods of time and analyzed by denaturing agarose gel electrophoresis. **L-N** FISH-Flow analysis of 28S rRNA in dTAG-treated C40 cells. Cells were treated with DMSO or dTAG for the indicated times, stained with DAPI and FISH probes targeting 28S rRNA, and analyzed by flow cytometry. Fluorescence intensities of stained or unstained (unst) cell populations are shown as histograms (**L**), contour plots (**M**), or as relative mean fluorescence intensities (MFIs) calculated by normalizing the geometric mean of 28S rRNA-Quasar670 intensity to DMSO-treated cells (**N**). Data in **N** are shown as mean ± SD (n = 4 biological replicates). *, $p = 0.0225$; **, $p < 0.0005$ (one-way ANOVA followed by Dunnett's multiple comparisons test). **O** As in **F**, northern analysis of the indicated tRNAs. Note that primary $tRNA^{Arg}_{UCU}$ transcripts contain an intron that is removed separately from their 5' leader and 3' trailer pre-tRNA sequences, yielding an additional band. See also Fig. 1H. m, mature tRNA; p, precursor tRNA (n = 3). **P** As in **F**, northern analysis of rRNA precursors using the northern probes mid (green) and 3' end (purple) shown in Fig. 1I (n = 3). Source data are provided as a Source Data file.

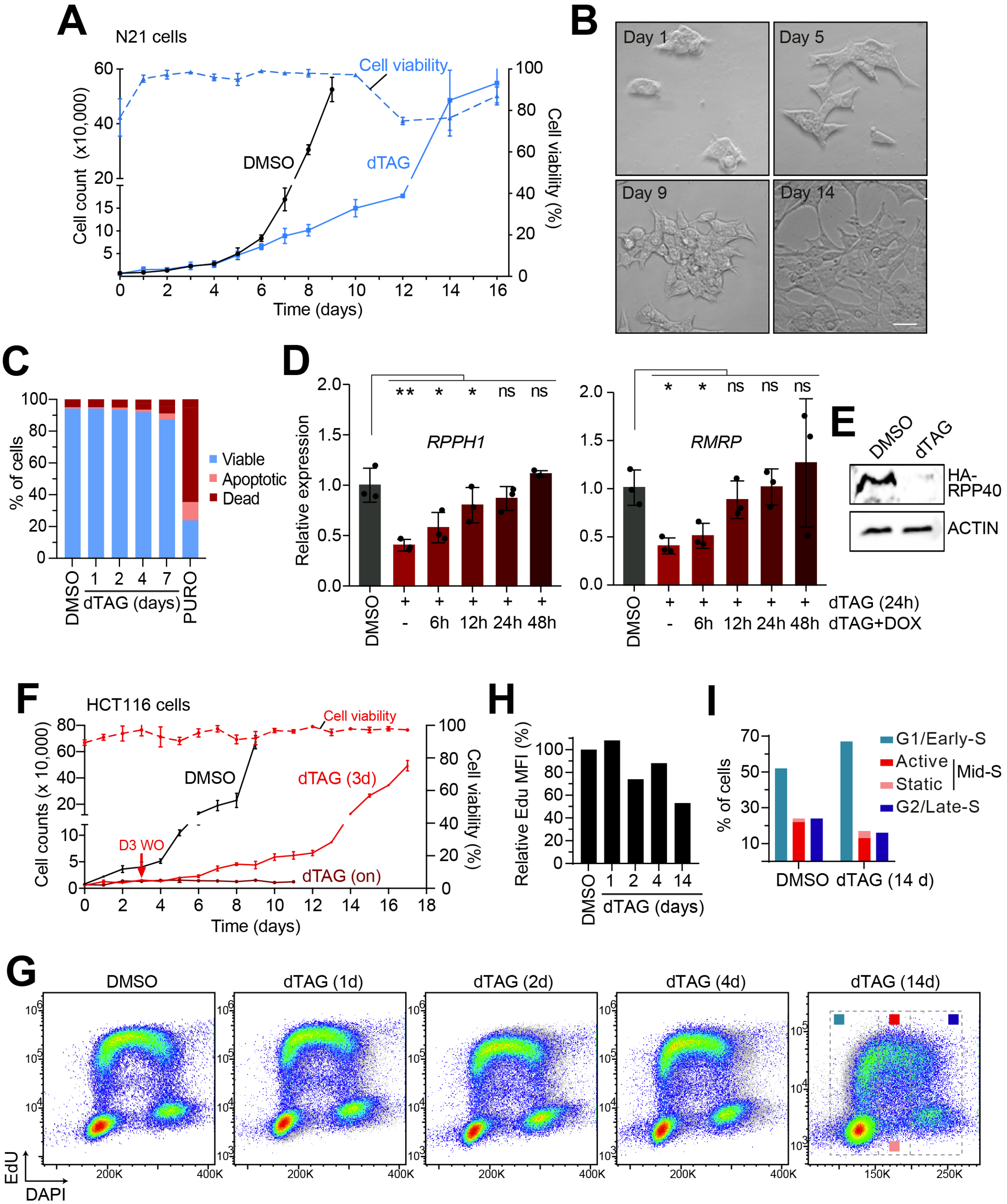

Supplementary Fig. 2 | Growth and viability of dTAG-treated N21 cells, C40 cells, Dox-inducible C40 cells, and modified HCT116. A Growth of N21 cells. Cells were treated either continuously with DMSO (black; control) or with dTAG (blue). Dashed blue line indicates

viability of the dTAG-treated N21 cells. Data are shown as mean ± SD (n = 3 biological replicates). **B** Representative brightfield views of N21 cells on the indicated days of treatment with dTAG. Scale bar, 30 µm. **C** Viability of dTAG-treated C40 cells analyzed by the Apoptosis Kit (ThermoFisher, Cat. nr. A35136). Cells were treated with dTAG for the indicated periods of time or with puromycin at 2 µg/ml for 12 h (PURO), co-stained with annexin V to detect externalized phosphatidylserine in apoptotic cells and SYTOX™ AADvanced™ stain to detect dead cells, and analyzed by flow cytometry. Proportions of viable, apoptotic, and dead cells were determined according to the manufacturer's instructions. **D** Relative quantification of *RPPH1* and *RMRP* by qPCR of RNA isolated from Dox-inducible C40 cells treated with DMSO or dTAG with or without Dox, as indicated (n = 3 biological replicates). Data are shown as mean ± SD. *, $p < 0.05$; **, $p = 0.0005$ (two-tailed Student's t test); ns, not significant; exact p values in Source Data file.. See also Fig. 2F, G. **E** Immunoblot analysis using an anti-HA antibody of lysates of HCT116 cells engineered for inducible degradation of endogenous RPP40, treated with DMSO or dTAG for 24 h (n = 3). **F** Growth of HCT116 cells engineered for inducible degradation of the endogenous RPP40. Cells were treated either continuously with DMSO (black; control), with dTAG for 3 days (red; red arrow indicates day 3 when dTAG was washed out, D3 WO), or continuously with dTAG (dark red, dTAG (on)). Dashed red line indicates viability of cells treated with dTAG for 3 days. Viability of cells treated continuously with dTAG was >90% at all analyzed time points (data not shown). Data are shown as mean ± SD (n = 3 biological replicates). **G-I** Cell cycle analysis of dTAG-treated N21 cells by flow cytometry. **G** Cells were treated with DMSO or dTAG for the indicated times, pulse-labeled with EdU and stained for the incorporated EdU (with Alexa Fluor 594, AF594) and DNA (with FxCycle Violet Stain), then analyzed by flow cytometry. **H** Relative mean fluorescence intensity (MFI) calculated by subtracting the mean EdU-AF594 intensity of unstained cells from the mean EdU-AF594 intensity of stained cells in Mid-S phase, gated as indicated in panel **G**, at the indicated times of DMSO or dTAG treatment. **I** Phases of the cell cycle quantified as indicated by the gating in **G** (dashed gray lines in the 14-day dot plot). Source data are provided as a Source Data file.

Supplementary Figure 3

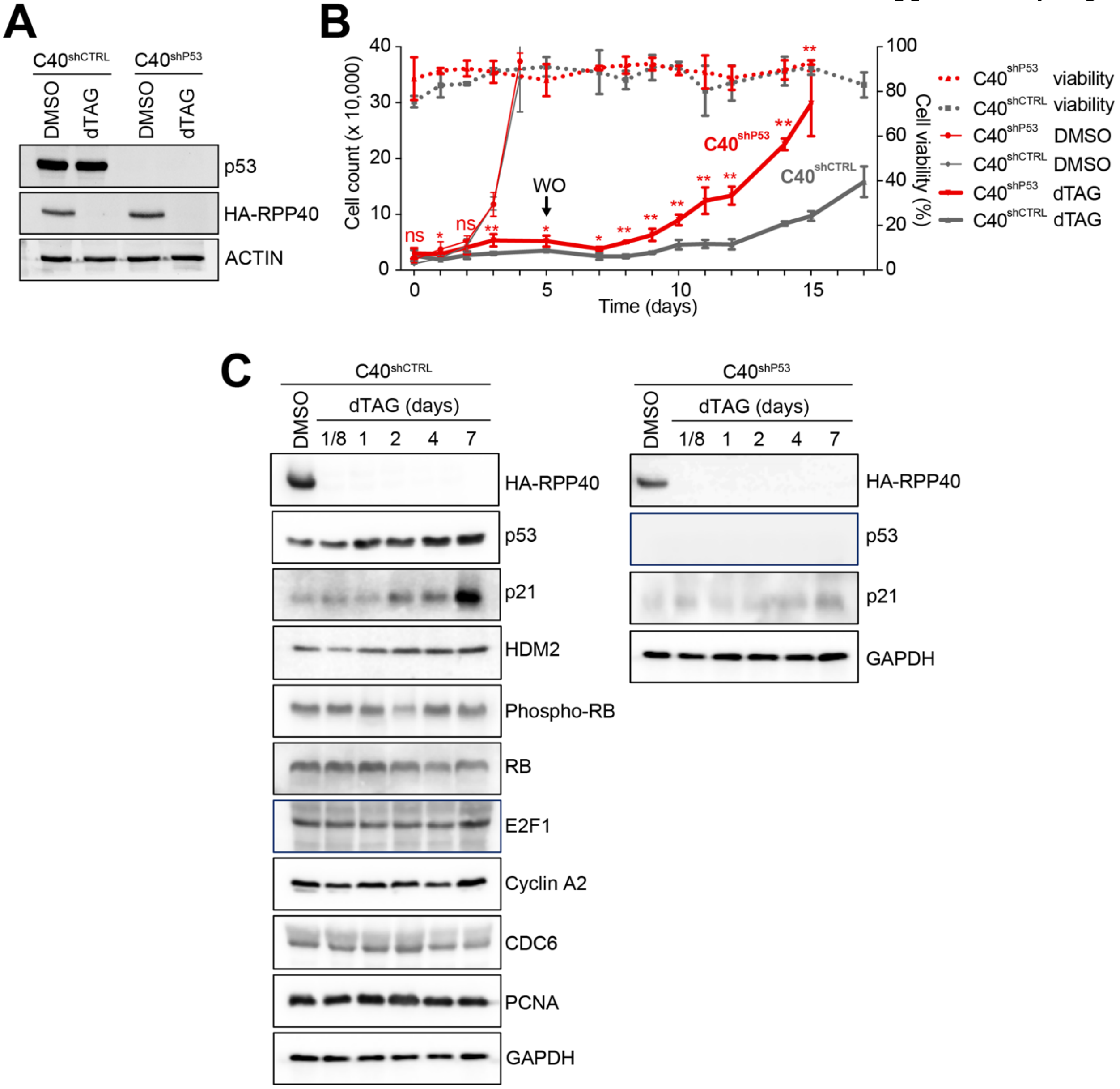

Supplementary Fig. 3 | Molecular characterization of the induced reversible proliferative arrest. A, B p53-independent induction of cellular a reversible proliferative arrest by rapid depletion of RNase MRP. **A** Immunoblot analysis of p53 and HA-tagged endogenous RPP40 in C40 cells stably expressing p53-targeting shRNA ($C40^{shP53}$) or non-targeting control shRNA ($C40^{shCTRL}$) treated with DMSO or dTAG for 24 h. Actin serves as a loading control (n = 3). Note that there was no obvious increase in p53 as a result of RPP40 depletion. **B** Growth of $C40^{shCTRL}$ (gray) and $C40^{shP53}$ (red) cells over the indicated periods of time. Cells were treated either continuously with DMSO (thin lines) or with dTAG for 5 days and then dTAG was washed out (WO; thick lines). Dotted lines indicate cell viability. Data are shown as mean ± SD (n = 3 biological replicates). *, $p < 0.05$; **, $p < 0.01$; exact p values in Source Data file; ns, non-significant (two-tailed Student's t test comparing $C40^{shP53}$ dTAG and $C40^{shCTRL}$ dTAG data). **C** Immunoblot analysis of factors associated with p53-dependent or independent response to

impaired ribosome biogenesis and/or regulation of cell cycle. C40^{shCTRL} or C40^{shP53} cells were analyzed at the indicated times of treatment with dTAG. Source data are provided as a Source Data file.

Supplementary Figure 4

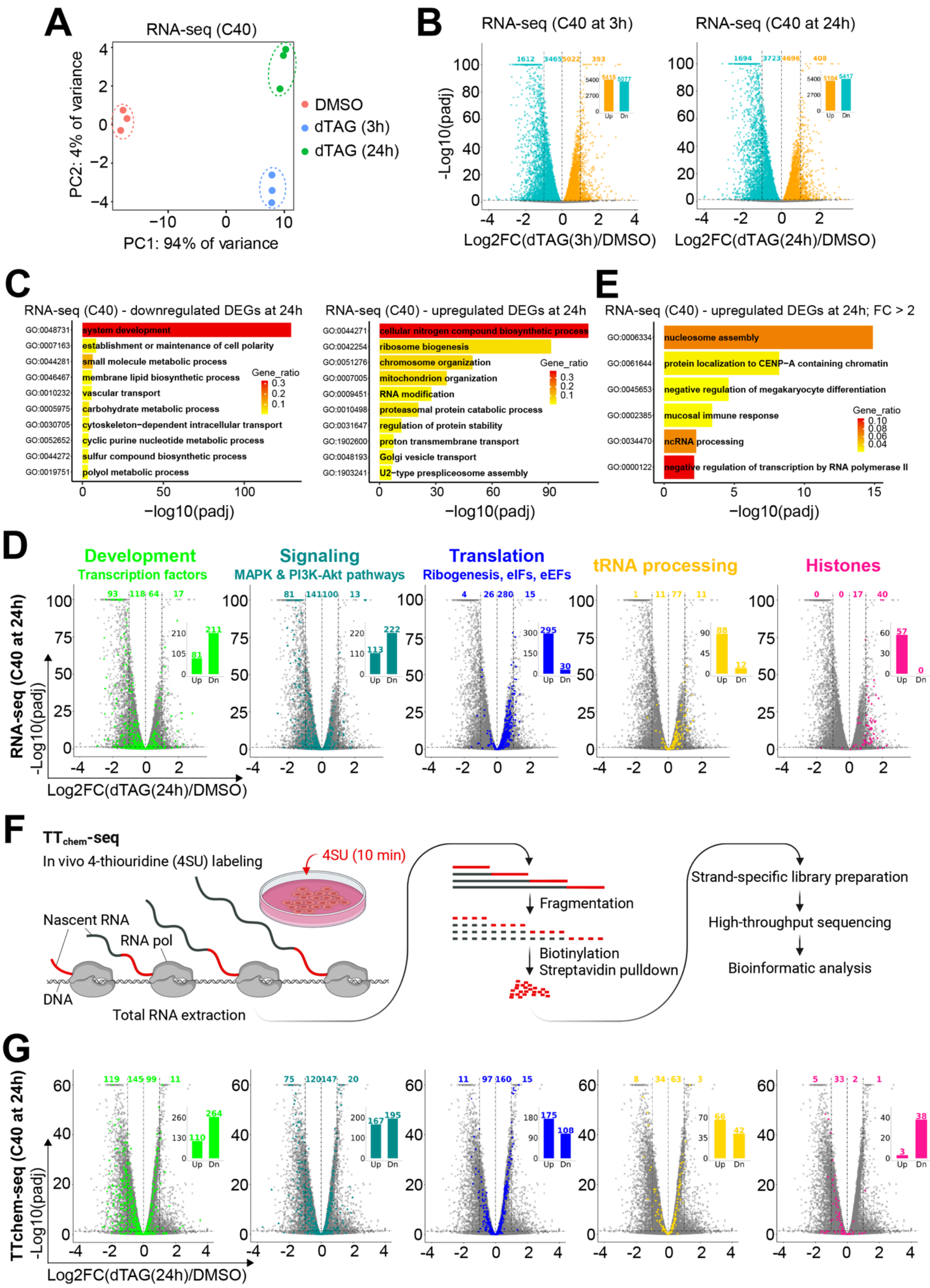

Supplementary Figure 4

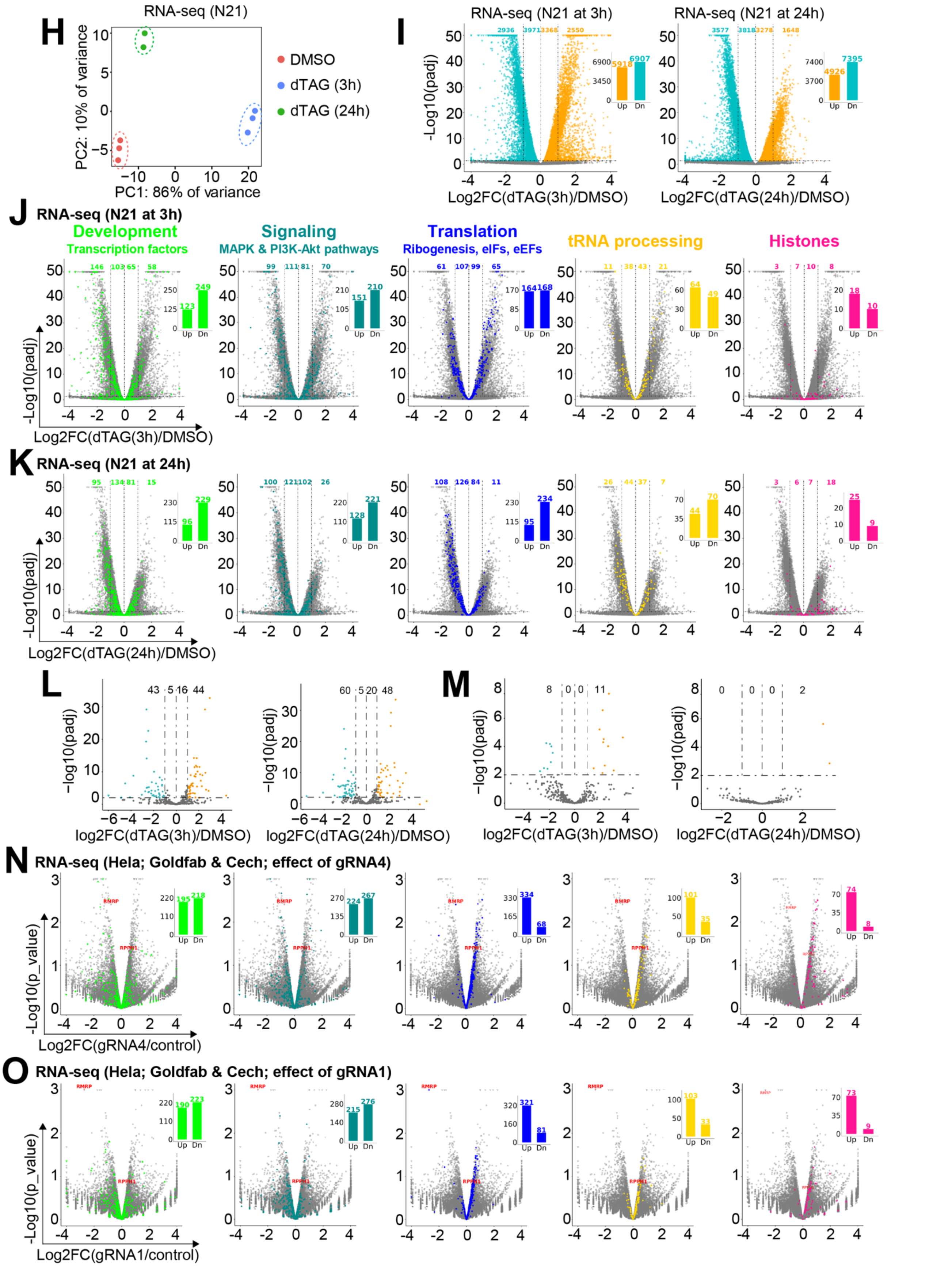

Supplementary Fig. 4 | Early transcriptional responses of human cells to depletion of RNases P and/or MRP. A PCA analysis of all C40 RNA-seq samples. Control samples are cells treated with DMSO for 24 h. Dashed ovals indicate clusters. **B** Volcano plots showing differential gene expression between C40 cells treated with DMSO or dTAG for 3 h (left) or 24 h (right; RNA-seq data; n = 3). Colored numbers above the volcano plots are counts of significantly regulated genes (adjusted p-value (padj) < 0.05) in different bins separated by dashed vertical lines according to the strength and sense of regulation. Insets summarize total numbers of significantly up- or downregulated genes. FC, fold change. **C** Top ten most significant Gene Ontology (GO) terms that are enriched among genes downregulated (left) or upregulated (right) following dTAG treatment for 24 h. See also **B** (right) and Supplementary Data 1. DEGs, differentially expressed genes. **D** Volcano plots showing differential gene expression for different functional categories of genes between C40 cells treated with DMSO or dTAG for 24 h (RNA-seq data; n = 3). Colored dots indicate genes belonging to the indicated functional categories (see also Supplementary Data 1). Colored numbers above the volcano plots are counts of significantly regulated color-highlighted genes (padj < 0.05) in different bins separated by dashed vertical lines according to the strength and sense of regulation. Insets summarize total numbers of significantly up- or downregulated color-highlighted genes. FC, fold change. See also Fig. 4A. **E** All GO terms that are enriched among genes upregulated by at least two-fold following dTAG treatment for 24 h. See also **D** and Supplementary Data 1. **F** Overview of the transient transcriptome sequencing with chemical RNA fragmentation (TT_{chem}-seq) method for profiling nascent transcription (adapted from Gregersen et al, Nat Protoc 2020)[2]. Nascent RNA is pulse-labeled by incubating cells in culture with 4-thiouridine (4SU) for 10 min. Total RNA is then extracted and fragmented by controlled base hydrolysis. The 4SU residues in the fragmented RNA (red) are biotinylated using a biotin linker and captured with streptavidin beads before strand-specific library preparation and high-throughput sequencing. RNA pol, RNA polymerase. Created in BioRender. Murn, J. (2025) https://BioRender.com/z11j170. **G** As in **D**, showing the impact of a 24-h dTAG treatment on nascent transcripts (TT_{chem}-seq data; n = 3). **H** PCA analysis of all N21 RNA-seq samples. Control samples are cells treated with DMSO for 24 h. Dashed ovals indicate clusters. **I-K** Acute transcriptional response of N21 cells to RNase P depletion. **I** As in **B**, but showing differential gene expression between N21 cells treated with DMSO or dTAG for 3 h (left) or 24 h (right; RNA-seq data; n = 3). **J, K** As in **I**, but highlighting in color genes belonging to the indicated functional categories (see also **D** and Supplementary Data 1). **L, M** Changes in tRNA transcription in response to rapid depletion of RNases P and MRP. **L** Volcano plots showing differential tRNA abundances between C40 cells treated with DMSO or dTAG for 3 h (left) or 24 h (right; TT_{chem}-seq data; n = 3). Numbers above the volcano plots are counts of significantly regulated tRNA transcripts (adjusted p-value (padj) ≤ 0.01) in different bins separated by dashed vertical lines according to the strength and sense of regulation. Transcripts showing twofold or larger changes in abundance are highlighted in color. FC, fold change. **M** As in **L**, but showing the results for N21 cells. The stronger perturbation of tRNA transcription in dTAG-treated C40 cells (in which both RNases P and MRP are depleted) compared to N21 cells (in which only RNase P is depleted) likely results from the potent regulatory effect of RNase MRP on cell metabolism, including transcription. **N, O** Analysis of total RNA-seq data reported by Goldfarb and Cech[3]. Volcano plots show differential gene expression between either of two composite *RMRP*-depleted HeLa cell populations (expressing the CRISPR guide 4 in **N** or guide 1 in **O**) and control HeLa cells expressing a non-targeting guide RNA (n = 2). Highlighted in color are the same groups of genes as in **D, G, J, K**.

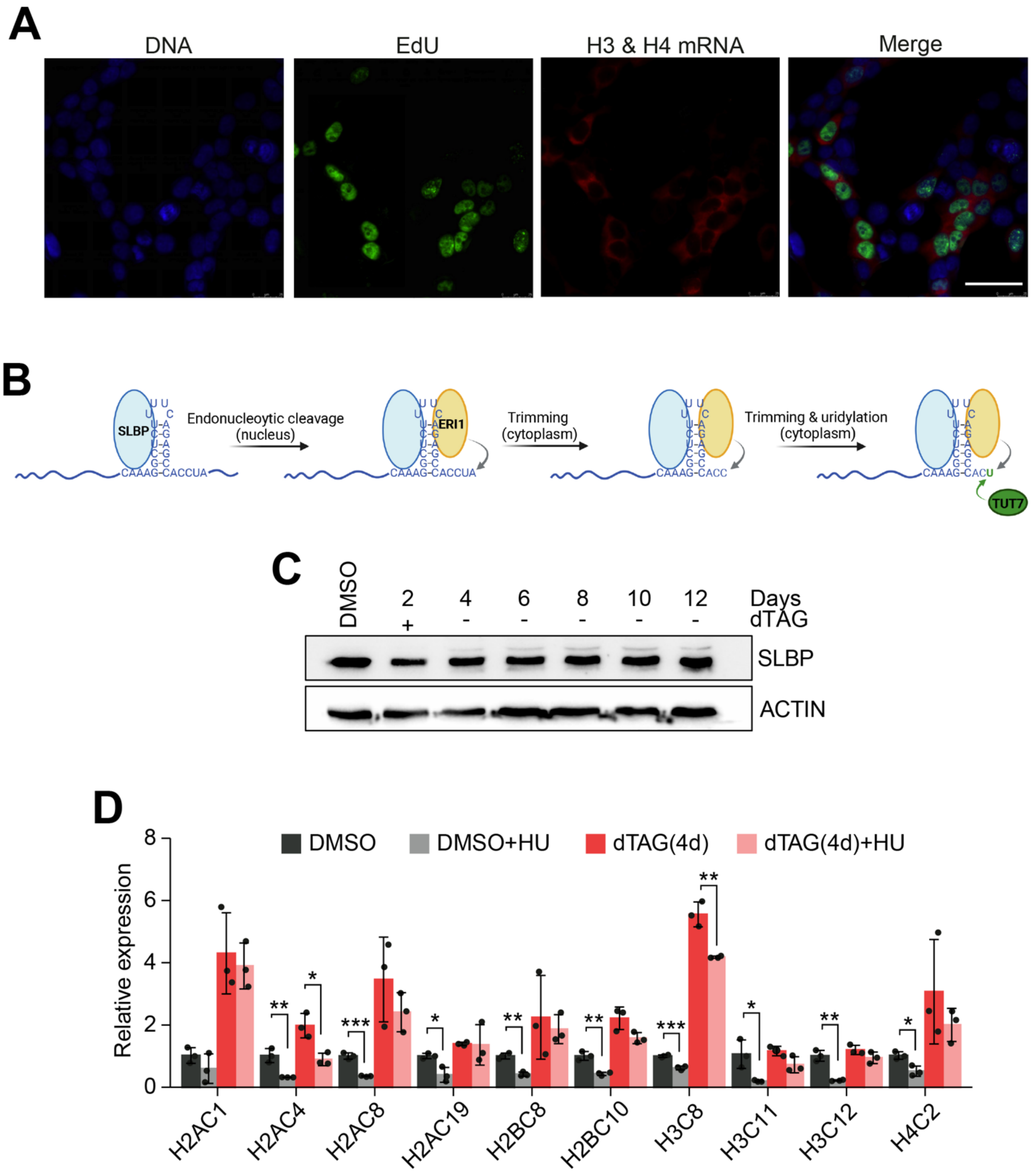

Supplementary Fig. 5 | Accumulation of histone mRNAs in cells lacking RNases P and MRP. A Representative image of C40 cells stained for DNA (blue), EdU (green), and histone H3 and H4 mRNAs (red). Scale bar, 50 µm. **B** Formation of a mature histone mRNA 3′ end requires endonucleolytic cleavage in the nucleus, which leaves a 5-nt tail after the stemloop, followed by trimming in the cytoplasm to leave a tail of 3 nts (ACC). If the tail is shortened to <3 nts, uridines are added by the terminal uridylyl transferase TUT7 to maintain the length of the

tail at 3 nts. Created in BioRender. Murn, J. (2025) https://BioRender.com/k72y969. **C** Time-course immunoblot analysis of SLBP in lysates of C40 cells treated with DMSO or dTAG for 3 days. ACTIN serves as a loading control (n = 3). See also Fig. 2A, D. **D** Relative quantification of the indicated histone mRNAs by qPCR in RNA isolated from C40 cells treated with DMSO or dTAG for 4 days prior to a 30-min treatment with hydroxyurea (HU). Data are shown as mean ± SD (n = 3 biological replicates). *, $p < 0.05$; **, $p < 0.01$; ***, $p < 0.005$ (two-tailed Student's t test); exact p values and source data in Source Data file.

Supplementary Figure 6

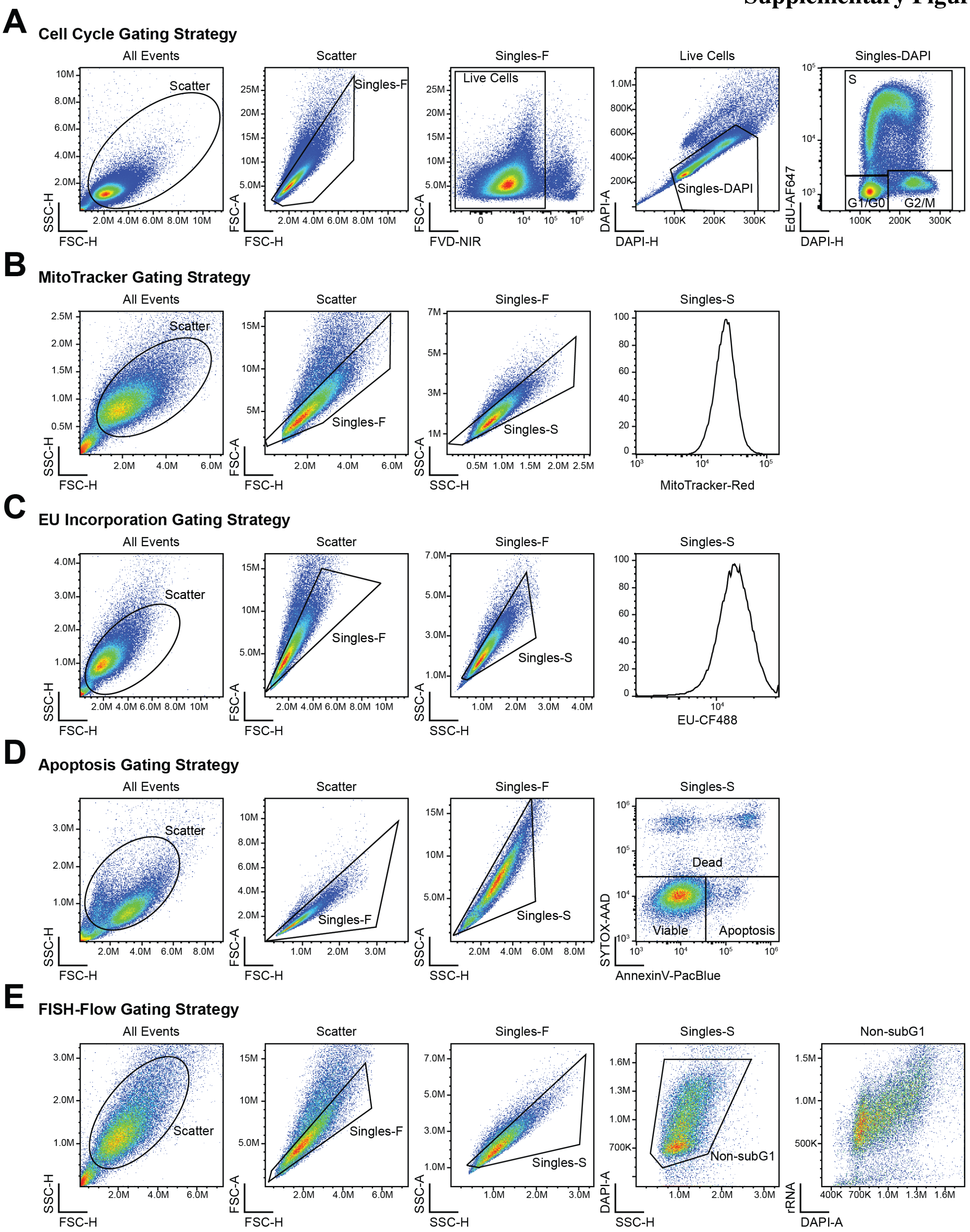

Supplementary Fig. 6 | Representative gating strategies for flow cytometry assays. A-C Untreated or DMSO-treated 293T cells. **D** C40 cells treated with dTAG for 7 d. **E** DMSO-treated C40 cells.

SUPPLEMENTARY REFERENCES

1. Nabet B*, et al.* The dTAG system for immediate and target-specific protein degradation. *Nat Chem Biol* **14**, 431-441 (2018).

2. Gregersen LH, Mitter R, Svejstrup JQ. Using TT(chem)-seq for profiling nascent transcription and measuring transcript elongation. *Nat Protoc* **15**, 604-627 (2020).

3. Goldfarb KC, Cech TR. Targeted CRISPR disruption reveals a role for RNase MRP RNA in human preribosomal RNA processing. *Genes Dev* **31**, 59-71 (2017).
