# Supplementary material for: Reversible proliferative arrest induced by rapid depletion of RNase MRP
Source: Nat Commun. 2025 Jun 18;16:5342. doi: 10.1038/s41467-025-60471-4 (PMC12177063; doi:10.1038/s41467-025-60471-4)
Supplement: Supplementary file 2 — Description of Additional Supplementary Files [file 41467_2025_60471_MOESM2_ESM.pdf]

Description of Additional Supplementary Files

File Name: Supplementary Data 1.

Description: Lists of protein-coding genes used for the functional annotation of differentially expressed transcripts in Fig. 4 and Supplementary Fig. 4.

File Name: Supplementary Data 2.

Description: Primers and oligonucleotide probes used in this study
