# Supplementary material for: Reversible proliferative arrest induced by rapid depletion of RNase MRP
Source: Nat Commun. 2025 Jun 18;16:5342. doi: 10.1038/s41467-025-60471-4 (PMC12177063; doi:10.1038/s41467-025-60471-4)
Supplement: Supplementary file 5 — Reporting Summary [file 41467_2025_60471_MOESM5_ESM.pdf]

nature portfolio

Corresponding author(s): Jernej Murn, William F. Marzluff, Sihem Cheloufi

Last updated by author(s): May 6, 2025

Reporting Summary

Nature Portfolio wishes to improve the reproducibility of the work that we publish. This form provides structure for consistency and transparency in reporting. For further information on Nature Portfolio policies, see our Editorial Policies and the Editorial Policy Checklist.

Statistics

For all statistical analyses, confirm that the following items are present in the figure legend, table legend, main text, or Methods section.

n/a	Confirmed	
☐	☒	The exact sample size (*n*) for each experimental group/condition, given as a discrete number and unit of measurement
☐	☒	A statement on whether measurements were taken from distinct samples or whether the same sample was measured repeatedly
☐	☒	The statistical test(s) used AND whether they are one- or two-sided<br>*Only common tests should be described solely by name; describe more complex techniques in the Methods section.*
☒	☐	A description of all covariates tested
☐	☒	A description of any assumptions or corrections, such as tests of normality and adjustment for multiple comparisons
☐	☒	A full description of the statistical parameters including central tendency (e.g. means) or other basic estimates (e.g. regression coefficient) AND variation (e.g. standard deviation) or associated estimates of uncertainty (e.g. confidence intervals)
☐	☒	For null hypothesis testing, the test statistic (e.g. *F*, *t*, *r*) with confidence intervals, effect sizes, degrees of freedom and *P* value noted<br>*Give P values as exact values whenever suitable.*
☒	☐	For Bayesian analysis, information on the choice of priors and Markov chain Monte Carlo settings
☒	☐	For hierarchical and complex designs, identification of the appropriate level for tests and full reporting of outcomes
☒	☐	Estimates of effect sizes (e.g. Cohen's *d*, Pearson's *r*), indicating how they were calculated

Our web collection on statistics for biologists contains articles on many of the points above.

Software and code

Policy information about availability of computer code

Data collection	Microscopy data was collected with LAS X (Leica) and ZEN (Zeiss) software. RT-qPCR data was collected with CFX Maestro (v2.2). Sequencing data was collected by Illumina Real-Time Analysis software. Northern data was collected using Amersham TYPHOON Control Software of Typhoon imager (Cytiva). Flow cytometry data was collected using NovoExpress (v1.5) on NovoCyte 2100Y/Quanteon cytometers. ATP production was measured using Wave Pro of Seahorse XF Pro analyzer (Agilent). Scans of gels and blots were performed using Image Lab Touch 3.0 Software of ChemiDOC imaging systems (Bio-Rad).
Data analysis	Data analysis was conducted with GraphPad Prism v9.2.0 or with R v3.6.2.

For manuscripts utilizing custom algorithms or software that are central to the research but not yet described in published literature, software must be made available to editors and reviewers. We strongly encourage code deposition in a community repository (e.g. GitHub). See the Nature Portfolio guidelines for submitting code & software for further information.

Data

Policy information about availability of data

All manuscripts must include a data availability statement. This statement should provide the following information, where applicable:

- Accession codes, unique identifiers, or web links for publicly available datasets
- A description of any restrictions on data availability
- For clinical datasets or third party data, please ensure that the statement adheres to our policy

The high-throughput sequencing data generated in this study have been deposited in the Gene Expression Omnibus (GEO) database under accession number GSE253620. Source data are provided with this paper.

Research involving human participants, their data, or biological material

Policy information about studies with human participants or human data. See also policy information about sex, gender (identity/presentation), and sexual orientation and race, ethnicity and racism.

Reporting on sex and gender	N/A
Reporting on race, ethnicity, or other socially relevant groupings	N/A
Population characteristics	N/A
Recruitment	N/A
Ethics oversight	N/A

Note that full information on the approval of the study protocol must also be provided in the manuscript.

Field-specific reporting

Please select the one below that is the best fit for your research. If you are not sure, read the appropriate sections before making your selection.

☒ Life sciences ☐ Behavioural & social sciences ☐ Ecological, evolutionary & environmental sciences

For a reference copy of the document with all sections, see nature.com/documents/nr-reporting-summary-flat.pdf

Life sciences study design

All studies must disclose on these points even when the disclosure is negative.

Sample size	Sample sizes were chosen to have enough samples for statistical analysis.
Data exclusions	Data were only excluded for failed experiments resulting from technical issues.
Replication	Experiments were repeated in triplicates to confirm experimental reproducibility. Multiple biological samples were tested to confirm biological reproducibility. All results reported in the manuscript have been reproduced in independent experiments.
Randomization	Cell samples were randomly allocated into experimental groups
Blinding	Blinding was not utilized due to objective data collection methods.

Reporting for specific materials, systems and methods

We require information from authors about some types of materials, experimental systems and methods used in many studies. Here, indicate whether each material, system or method listed is relevant to your study. If you are not sure if a list item applies to your research, read the appropriate section before selecting a response.

Materials & experimental systems

n/a	Involved in the study
☐	☒ Antibodies
☐	☒ Eukaryotic cell lines
☒	☐ Palaeontology and archaeology
☒	☐ Animals and other organisms
☒	☐ Clinical data
☒	☐ Dual use research of concern
☒	☐ Plants

Methods

n/a	Involved in the study
☒	☐ ChIP-seq
☐	☒ Flow cytometry
☒	☐ MRI-based neuroimaging

Antibodies

Antibodies used

POP1 (Abcam, ab254978)
POP5 (Santa Cruz Biotechnology, sc-23046),
RPP14 (Thermo Fisher Scientific, PA5-57567)
RPP20 (Santa Cruz Biotechnology, sc-244043)
RPP21 (Proteintech, 16377-1-AP)
RPP25 (Sigma, PA046900-100UL)
RPP29 (Santa Cruz Biotechnology, sc-23048)
RPP30 (Santa Cruz Biotechnology, sc-81374)
RPP38 (Santa Cruz Biotechnology, sc-398113)
RPP40 (Proteintech, 11736-1-AP)
E2F1 (Santa Cruz Biotechnology, sc-251)
SLBP (Thermo Fisher Scientific, PA5-66410),
FLAG M2 (Millipore Sigma, F1804-200UG),
Puromycin (Millipore, MABE343),
Cyclin A2 (Cell Signaling Technology,4656),
phospho-Rb (Ser807/811) (Cell Signaling Technology, 9308),
Rb (BD Biosciences, 554136),
p21 (Cell Signaling Technology, 2947),
PCNA (Cell Signaling Technology, 13110),
CDC6 (Abcam, ab188423),
β-Actin–Peroxidase antibody (Millipore Sigma, A3854)
p53/pab122, gift of Yanping Zhang (UNC); PMID: 25117711
MDM2/2A10, gift of Yanping Zhang (UNC); PMID: 25117711

All antibodies used for immunoblotting were used at 1:1,000 dilution, except p53/pab122 and MDM2/2A10, which were used at 1:30 dilution, and β-Actin–Peroxidase antibody, which was used at 1:25,000, as recommended by the manufacturer. For immunofluorescence, antibodies were used at 1:200 dilution.

Validation

The above antibodies have been validated by the manufacturer as follows (antibody, species, validated for application):
POP1, human ICC/IF
RPP14, human WB
RPP20, human ELISA, WB
RPP21, human WB
RPP25, human WB, IHC, IF
RPP29, human WB
RPP30, human WB, IP
RPP38, human WB, IP, IF and ELISA
RPP40, human WB
E2F1, human WB, IP, IF and IHC(P)
SLBP, human WB, IF
Flag, IF and WB
Puromycin, human FACS, IF, ICC, WB, IP, IHC
Cyclin A2, human WB
phospho-Rb (Ser807/811), human WB, IP
Rb, human WB, IF
p21, human WB, IP, IHC, IF, Flow cytometry
PCNA, human WB, IP, IHC, IF, Flow
CDC6, human IP, WB, ICC/IF
β-Actin–Peroxidase antibody, human WB, IP

Mouse anti-p53/pab122 and mouse anti-MDM2/2A10 have been validated by Y. Zhang and coworkers (PMID: 25117711).

Eukaryotic cell lines

Policy information about cell lines and Sex and Gender in Research

Cell line source(s)	293T cells, ATCC (CRL-3216); HCT116 cells, ATCC (CCL-247).
Authentication	Cells were authenticated by ATCC using STR profiling.
Mycoplasma contamination	Cell lines routinely tested negative for mycoplasma.
Commonly misidentified lines (See ICLAC register)	None of the cell lines used in this study are listed in the ICLAC register.

Plants

Seed stocks	*Report on the source of all seed stocks or other plant material used. If applicable, state the seed stock centre and catalogue number. If plant specimens were collected from the field, describe the collection location, date and sampling procedures.*
Novel plant genotypes	*Describe the methods by which all novel plant genotypes were produced. This includes those generated by transgenic approaches, gene editing, chemical/radiation-based mutagenesis and hybridization. For transgenic lines, describe the transformation method, the number of independent lines analyzed and the generation upon which experiments were performed. For gene-edited lines, describe the editor used, the endogenous sequence targeted for editing, the targeting guide RNA sequence (if applicable) and how the editor was applied.*
Authentication	*Describe any authentication procedures for each seed stock used or novel genotype generated. Describe any experiments used to assess the effect of a mutation and, where applicable, how potential secondary effects (e.g. second site T-DNA insertions, mosiacism, off-target gene editing) were examined.*

Flow Cytometry

Plots

Confirm that:

☒ The axis labels state the marker and fluorochrome used (e.g. CD4-FITC).

☒ The axis scales are clearly visible. Include numbers along axes only for bottom left plot of group (a 'group' is an analysis of identical markers).

☒ All plots are contour plots with outliers or pseudocolor plots.

☒ A numerical value for number of cells or percentage (with statistics) is provided.

Methodology

Sample preparation	Samples were prepared as described in the Methods section. Cells were trypsinized, washed with DBPS or FACS buffer (5% FBS + 1mM EDTA in DPBS) and strained to ensure single cell suspension before analyzing by flow cytometry. Detailed, assay-specific preparations are provided in the Methods section.
Instrument	Agilent Novocyte 2100YB, Novocyte Quanteon
Software	Flow data was collected with NovoExpress (v1.5.6). Analysis was perfomed with FlowJo (v10.x)
Cell population abundance	Experiments are run on cultured cell lines, so relative population abundance and purity was not determined. At least 10,000 events were analyzed for each run.
Gating strategy	Forward vs side scatter gating is used to exclude debris, indicated by low forward and side scatter values, and dead cells, indicated by high side to forward scatter ratios. For the apoptosis assay, dying cells were included in the initial gate. Single cells were gated using height versus area ratios, gating out cells with unusually high area to height ratio. For cells stained with viability dyes, negative cells were gated as viable/live cells. Assay specific gates are shown in the representative gating strategy figure. Cell cycle gates were determined by analyzing the classic DNA content profile with a G1/G0 peak considered as 2n DNA content, G2/M as cells with 4n DNA, and EdU+ cells as S. DNA content was also gated to only include cells within the major DNA content distribution.

☒ Tick this box to confirm that a figure exemplifying the gating strategy is provided in the Supplementary Information.
